# Variations in the *NBN/NBS1 *gene and the risk of breast cancer in non-*BRCA1/2 *French Canadian families with high risk of breast cancer

**DOI:** 10.1186/1471-2407-9-181

**Published:** 2009-06-12

**Authors:** Sylvie Desjardins, Joly Charles Beauparlant, Yvan Labrie, Geneviève Ouellette, INHERIT BRCAs, Francine Durocher

**Affiliations:** 1 Cancer Genomics Laboratory, Oncology and Molecular Endocrinology Research Centre, Centre Hospitalier Universitaire de Québec and Laval University, Québec, Canada

## Abstract

**Background:**

The Nijmegen Breakage Syndrome is a chromosomal instability disorder characterized by microcephaly, growth retardation, immunodeficiency, and increased frequency of cancers. Familial studies on relatives of these patients indicated that they also appear to be at increased risk of cancer.

**Methods:**

In a candidate gene study aiming at identifying genetic determinants of breast cancer susceptibility, we undertook the full sequencing of the *NBN *gene in our cohort of 97 high-risk non-*BRCA1 *and -*BRCA2 *breast cancer families, along with 74 healthy unrelated controls, also from the French Canadian population. *In silico *programs (ESEfinder, NNSplice, Splice Site Finder and MatInspector) were used to assess the putative impact of the variants identified. The effect of the promoter variant was further studied by luciferase gene reporter assay in MCF-7, HEK293, HeLa and LNCaP cell lines.

**Results:**

Twenty-four variants were identified in our case series and their frequency was further evaluated in healthy controls. The potentially deleterious p.Ile171Val variant was observed in one case only. The p.Arg215Trp variant, suggested to impair NBN binding to histone γ-H2AX, was observed in one breast cancer case and one healthy control. A promoter variant c.-242-110delAGTA displayed a significant variation in frequency between both sample sets. Luciferase reporter gene assay of the promoter construct bearing this variant did not suggest a variation of expression in the MCF-7 breast cancer cell line, but indicated a reduction of luciferase expression in both the HEK293 and LNCaP cell lines.

**Conclusion:**

Our analysis of *NBN *sequence variations indicated that potential *NBN *alterations are present, albeit at a low frequency, in our cohort of high-risk breast cancer cases. Further analyses will be needed to fully ascertain the exact impact of those variants on breast cancer susceptibility, in particular for variants located in *NBN *promoter region.

## Background

Pathogenic mutations in *BRCA1*, *BRCA2*, *TP53*, *ATM*, *CHEK2*, *BRIP1 *and *PALB2 *have been associated with an increased breast cancer risk and, together, are found in less than 25% of breast cancer families showing a clear pattern of inheritance (high-risk families) [1]. It is thus clear that other susceptibility alleles remain to be identified to explain the increased risk in the remnant high-risk families. As the number and characteristics of such alleles are undetermined, a focussed candidate gene approach based on genes closely interacting with the known susceptibility genes such as *BRCA1 *and *BRCA2*, the two major susceptibility genes identified yet, constitutes a study design of choice to identify rare-moderate-penetrance susceptibility alleles.

In the cell, nibrin, encoded by the *NBN *gene (also known as *NBS1*), participates in pathways of double strand breaks (DSB)-induced DNA repair and, together with its partners MRE11A and RAD50, is required for activation of these pathways in response to DNA damages [2]. In fact, nibrin is at the crossroad of several pathways implicating genes already associated with breast cancer susceptibility and/or chromosomal instability disorders [2,3]. Individuals homozygous for hypomorphic mutations in *NBN *suffer from the Nijmegen Breakage Syndrome (NBS), an autosomal recessive chromosomal instability disorder characterized by microcephaly, growth retardation, immunodeficiency and hyper-radiosensitivity [4]. Cancers, in particular haematological malignancies, are common adverse events in patients with NBS, as almost 40% of them develop a malignancy before the age of 21 years, and this correlates with a marked impairment in DSB repair observed in cells from these patients [5].

Some studies have associated an heterozygous *NBN *status with numerous types of cancers, including breast cancer [6-10], suggesting that being a carrier of a deleterious mutation in *NBN *may confer an increased risk of approximately 2 to 3-fold [6]. This was also supported by the observation that relatives of NBS patients display a higher than expected rate of cancers [4,11]. However, other studies failed to find an association with an increased risk of cancer [12,13].

In support of a role of *NBN *in tumor formation, evidence from mouse models demonstrated that *Nbn *heterozygosity predisposes cells to malignancies, as they display a wide variety of tumors: liver, mammary gland, prostate, lung as well as lymphomas [14]. Indeed, cells from these mice displayed an elevated frequency of chromosomal aberrations. These observations were correlated by studies of *NBN *heterozygous mutation carriers demonstrating that cell lines from these individuals showed spontaneous chromosomal instability (chromatid and chromosomes breaks, and chromosomes rearrangements) [15,16] as well as increased sensitivity to radiation-induced chromosomal aberrations [17]. Thus, it has been hypothesized that in cells of carriers of deleterious mutations in DNA repair genes such as *NBN*, a decrease in DNA repair capabilities resulting from a gene dosage effect (i.e. lower gene expression) may be sufficient to create a permissive environment for tumor development [18,19]. It has also been suggested that these DNA repair genes may show differences in tissue-specific protein-dosage thresholds, below which they may fail to operate normally [20].

Thus, based on the close relation of *NBN *and known breast cancer susceptibility genes in the cell DNA repair pathways, and the studies suggesting a possible involvement of *NBN *alterations in cancer susceptibility, we undertook the analysis of the entire coding sequence, intron/exon junctions, as well as the proximal promoter region of the *NBN *gene. We therefore performed a thorough re-sequencing of a series of 97 breast cancer cases selected from high-risk families from the French Canadian population, and 74 unrelated healthy controls from the same origin for sequence variations that could possibly modulate breast cancer risk.

## Methods

### Ascertainment of families

All 97 non-*BRCA1/2 *individuals from high-risk French Canadian breast and ovarian cancer families participating in this study were part of a larger interdisciplinary program termed INHERIT BRCAs [21]. All participants were at least 18 years of age and had to sign an informed consent form. Ethics committees reviewed the research project at the 7 participating institutions from which the patients were referred. The details regarding selection criteria of the breast cancer cases as well as the experimental and clinical procedures have been described previously [21,22].

### PCR amplification and direct sequencing

PCR amplification of *NBN *(NM_002485.4) coding sequence, as well as flanking intronic regions, was performed on breast cancer cases and controls using primer pairs as described in Table 1. Sequencing reactions and sequence analysis were performed as described previously [22]. Alternative splice screening was also performed on cDNA on a subset of breast cancer cases using primers as described in Table 1.

**Table 1:** Oligonucleotides used for *NBN *PCR amplification and sequencing.

	F/R^1^	Oligonucleotide sequence (5'→3')	Annealing temp. (°C)	PCR lenght (bp)	MgCl_2 _final conc. (mM)	Comments
Genomic sequence						

Exon 1	F	GACGTTAAGACAAGTTGATTTGAACTTAGA*	60	965/	0.5	2% DMSO added to the PCR reaction
	R	TCCGCCCATGCTAACTTCCT		1113		
	F^2^	TTTAGTAGTGCGCAGGATGTAGAC				

Exon 2	F	CCTTTGATAGCCTTCAGTGAGGC	64	743	1.0	Sequencing primer:
	R	AGCCAGAGTCATGAAGGTCTGTTC				CCACTGGTACCACTGCCACA

Exon 3	F	GTCAGGAGAATCCCCACTGACTT*	60	548	1.5	
	R	GGCACAGAGTCCAATACTGTGCT				

Exon 4	F	TGGGAAGTTACATTTCTTCGATTCC*	58	728	1.5	
	R	GCACTGTCATAACCTTCTCGGTG				

Exon 5	F	GCAGTGACCAAAGACCGACTTCTA*	58	561	1.5	
	R	TGAGGTTACCTCAGTGCCATTTACT*				

Exon 6	F	AAACGCGATTAGATGCTTTTTGTC*	60	630	1.5	
	R	ACCCCACTTTGGTACACAGAAC				

Exon 7	F	CCACAGAGAGTGTAACAGTTCCAGG*	63	1100	1.0	2% DMSO added to the PCR reaction
	R	TTAATTCTTGTATCGGCCGGG				

Exon 8	F	TAACAGTGCCCCAGCGAGTAAG*	58	785	1.5	
	R	TCCTCTTACACTGTCGACCCTTAGA				

Exon 9	F	TTAGATAAGCCCGTCATAGATGCC*	58	516	1.5	
	R	TAACTACTCGCCGCTCCTTTACA				

Exon 10	F	GTTTGTCAGTCGTCTATAGTGGAGCA*	58	763	1.5	
	R	AATTGCGGCAAGTAAAATAACACG				

Exon 11	F	CCCTGCCCACAACCTTACTACG*	60	1500	1.5	
	R	GACCACAGCCATGAATGAGTGG *				

Exon 12	F	TCCTACCATCTACAGACAACCATGG	58	619	1.5	
	R	CCATGATCAATCCATTTCAAGGC *				

Exon 13	F	GCAAACAGTGCTGAGATTTTGTGTC	58	850	1.5	
	R	CCTGAGCTAAAGAACCTCCTCAAGTAG *				

Exon 14	F	CAACATCTCCTGCTTGGACTCTG	60	638	1.0	Sequencing primer:
	R	GAAGAATTTGCTTGAAGGCCACC				GATGGGTTTAGAACAGAGTTACTGCT

Exon 15	F	GAACTCAGATGTGGTGACCTCCAG	58	441	1.5	
	R	CAATTTCACACAATTCGGGAACC *				

Exon 16	F	GAGGAATGGGGATCTTTGAAGC	58	457	1.5	Sequencing primer:
	R	GTAACTTAAATCGCTTCTATACAC				AAGCAACATCAAAGGGATACATGA

cDNA						

PCR 1	F	GTTACGCGGTTGCACGTCG	64	998	1.5	Exons 1 to 8
	R	GTCATGAAAATCACCGCCAATC				

PCR 2	F	TCTTTTTGGCTCCGGGAACG	62	567	1.5	Exons 7 to 10
	R	GCTGCTGCTGAGAAGCCCTATC				Overlap of 169 pb with PCR1

PCR 3	F	CCCACTGTAAAGGAGTCCTGCA	62	634	1.5	Exons 10 to 12
	R	TACTTTCTGGTACTGCTTCATCACT				Overlap of 151 pb with PCR2

PCR 4	F	CCATAGAAGATGAAGTATTGGA	62	700	1.5	Exons 11 to 16
	R	GTAACTTAAATCGCTTCTATACAC				Overlap of 165 pb with PCR3

Promoter cloning						

	F	GACGACGCTAGCGACGTTAAGACAAGTTGATTTGAACTTAGA	62	515	1.0	2% DMSO added to the PCR reaction Added restriction sites are underlined
	R	GACGACAAGCTTATCGGTCCGGCTCCTCAGGGCTG				

^1 ^Primer direction: forward (F) or reverse (R) ^2 ^Alternative primer Oligonucleotides used as sequencing primers are marked with an (*)

### Variants characterization and haplotype estimations

Deviation from Hardy-Weinberg equilibrium (HWE) and allelic difference between both series was evaluated using a two-sided Chi Square test with 1 degree of freedom. The possible effect of a given variant on exonic splicing enhancers was assessed using the ESEfinder 3.0 program [23] and on splice consensus sites using NNSPLICE [24] and Splice Site Finder [25] web based programs. The potential impact of the promoter variant and pairwise linkage disequilibrium were evaluated as described elsewhere [26]. Haplotype analysis was performed using the WHAP program [27] implementing a regression-based association test allowing for evaluation of haplotype-specific association. Haplotype analyses were performed on all variants identified as well as on variants showing a minor allele frequency (MAF) greater than 5%.

### Luciferase promoter assays

A portion of 401 bp of the *NBN *promoter region, and the entire 5'UTR sequence (110 bp), was amplified by PCR from a breast cancer individual carrier of the c.-242-110delAGTA deletion using primers introducing a *NheI *or a *HindIII *restriction site (Table 1). PCR products were then digested and introduced in the pGL3 basic vector (Promega Corporation, Madison, WI, USA). Direct sequencing of clones was performed to confirm the presence of the reference sequence allele as well as the four nucleotides deletion. Transient transfections in MCF-7, LNCaP, HeLa and HEK293 cells and dual luciferase reporter assays were performed as described previously [26], with cells harvested 24–48 h after transfection. *ATBF1 *expression levels were measured in these four cell lines by quantitative real-time PCR (QRT-PCR) as described previously [28], using RNA extracted by the TriReagent method (Molecular Research Center Inc, Cincinnati, OH, USA) and specific primers (sens: 5'-TGCAACTAAACCGCCCACATATA-3'; antisens 5'-CCCCAAGTGAGATAAAGCTAAACAAA-3'). Levels of expression were normalized using the housekeeping gene *HPRT1*, and are indicated relative to the MCF-7 cell line (sens primer: 5'-AGTTCTGTGGCCATCTGCTTAGTAG-3'; antisens primer: 5'-AAACAACAATCCGCCCAAAGG-3').

## Results

### Sequence variations in the NBN gene

**Figure 1:**
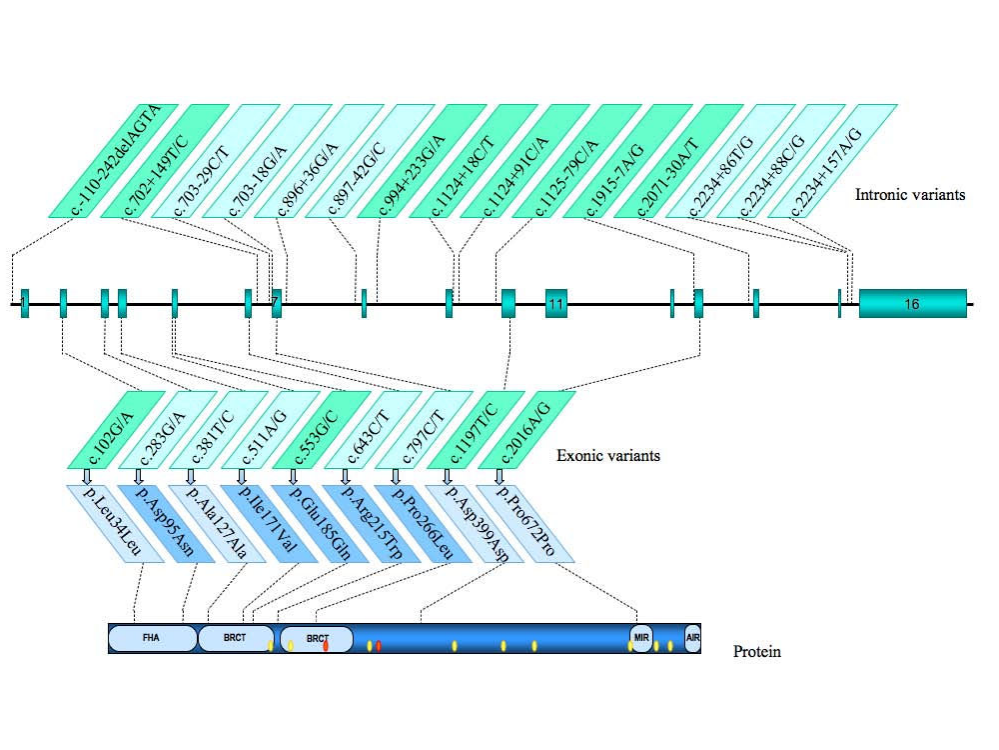
**Schematic representation of the *NBN *gene on chromosome 8q21**. The upper part shows variants identified in the *NBN *gene. Light green boxes refer to SNPs with MAF ≥ 5% in the cases set, and variants name are indicated according to the reference sequence NM_002485.4. Corresponding coding variations are indicated relative to the protein structure in the bottom part of the figure. Light blue boxes refer to synonymous variations. FHA: Forkhead-associated domain. BRCT: BRCA1 C-terminal domain. MIR: MRE11 interacting region. AIR: ATM interacting region. Yellow dots: acetylated lysine residues (K208, K233, K334, K441, K504, K544, K665, K690, K698 and K715). Red dots: phosphorylated serine residues (S278 and S343).

Direct sequencing of *NBN *entire coding region, adjacent intronic sequences as well as the proximal promoter region was performed on 97 affected individuals from French Canadian breast and ovarian cancer families (one individual per family). Twenty-four variants were identified (Tables 2, 3 and Figure 1), including a new rare synonymous change at codon 127 (c.381T/C) and a new variant located in intron 15 (c.2234+86T/G), both not reported in the literature and databases, and found exclusively among breast cancer cases. Variants identified in the case dataset were also genotyped in 74 healthy individuals from the same population. All SNPs genotyped were in HWE. Nine out of the 24 variations were located in the coding region (Table 2), while the remaining 15 were located in untranslated regions (Table 3). Among all variants identified, half (12) were considered as common (MAF ≥ 5%) while the remaining 12 were rare variations. Two rare non-synonymous exonic variants (c.511A/G and c.797C/T) were observed only once in the case series, of which p.Ile171Val is located in the first BRCA1 C-terminal (BRCT) domain (Figure 1). Of these two variants, only c.511A/G could be genotyped in an additional family member, i.e. an unaffected male cousin, who was found non-carrier (data not shown). Two other rare variants, c.283G/A and c.643C/T, were both observed in one breast cancer case and one control. For the c.643C/T variant, a DNA sample from another available family member affected with ovarian cancer was analyzed and turned out to be wild type (data not shown). Among the variants located in untranslated regions (Table 3), the c.2234+86T/G variant was present exclusively in two breast cancer cases. When genotype frequencies of all variants were compared between both series, only the c.-242-110delAGTA variant in the promoter showed a statistically significant association with breast cancer (OR 3.4, 95% CI: 1.1–10.5; p = 0.029).

**Table 2:** Exonic sequence variations of the NBN gene in French Canadian breast cancer cases and healthy controls.

SNP	SNP ID^1 ^(dbSNP ID)	Protein change	Location	Series (No.)	MAF^2^	Common homozygote No. (expected)^3^	Heterozygote No. (expected)^3^	Rare homozygote No. (expected)^3^	HWE p-value	OR (95% CI)^4^	Reported MAF in Caucasian
1	c.102G/A	p.Leu34Leu	Exon 2	Cases (97)	0.284	52 (49.80)	35 (39.41)	10 (7.80)	0.27	0.7	0.283–0.450^5^
	(rs1063045)			Controls (71)	0.310	33 (33.82)	32 (30.37)	6 (6.82)	0.65	(0.4–1.3)	

2	c.283G/A	p.Asp95Asn	Exon 3	Cases (97)	0.005	96 (96.00)	1 (0.99)	0 (0)	0.96	0.7	
	(NA)^6^			Controls (71)	0.007	70 (70.00)	1 (0.99)	0 (0)	0.95	(0.0–11.9)	

3	c.381T/C	p.Ala127Ala	Exon 4	Cases (97)	0.005	96 (96.00)	1 (0.99)	0 (0)	0.96	2.3	
	(NA)			Controls (72)	0.000	72 (72.00)	0 (0)	0 (0)	1.00	(0.1–56.1)	

4	c.511A/G	p.Ile171Val	Exon 5	Cases (97)	0.005	96 (96.00)	1 (0.99)	0 (0)	0.96	2.3	
	(NA)^6^			Controls (73)	0.000	73 (73.00)	0 (0)	0 (0)	1.00	(0.1–57.0)	

5	c.553G/C	p.Glu185Gln	Exon 5	Cases (97)	0.268	54 (51.97)	34 (38.06)	9 (6.97)	0.29	0.6	
	(rs1805794)			Controls (73)	0.308	34 (34.93)	33 (31.13)	6 (6.93)	0.61	(0.3–1.2)	

6	c.643C/T	p.Arg215Trp	Exon 6	Cases (97)	0.005	96 (96.00)	1 (0.99)	0 (0)	0.96	0.8	
	(rs34767364)			Controls (73)	0.007	72 (72.00)	1 (0.99)	0 (0)	0.95	(0.0–12.2)	

7	c.797C/T	p.Pro266Leu	Exon 7	Cases (97)	0.005	96 (96.00)	1 (0.99)	0 (0)	0.96	2.3	0.000^5^
	(rs769420)			Controls (73)	0.000	73 (73.00)	0 (0)	0 (0)	1.00	(0.1–57.0)	

8	c.1197T/C	p.Asp399Asp	Exon 10	Cases (97)	0.320	47 (44.91)	38 (42.19)	12 (9.91)	0.33	0.9	0.28–0.45^5^
	(rs709816)			Controls (72)	0.313	34 (34.03)	31 (30.94)	7 (7.03)	0.99	(0.5–1.7)	

9	c.2016A/G	p.Pro672Pro	Exon 13	Cases (97)	0.284	52 (49.80)	35 (39.41)	10 (7.80)	0.27	0.8	0.283^5^
	(rs1061302)			Controls (70)	0.300	34 (34.30)	30 (29.40)	6 (6.30)	0.86	(0.4–2.2)	

^1 ^According to the nomenclature guidelines of the Human Genome Variation Society (reference sequence NM_002485.4), nucleotide number one being the A from the ATG codon.

^2 ^Minor allele frequency

^3 ^As expected under Hardy-Weinberg equilibrium

^4 ^Odds ratios for comparison of heterozygotes versus common homozygotes

^5 ^From NCBI dbSNP data.

^6 ^No entry in NCBI dbSNP database, although reported in the literature.

**Table 3:** Promoter and intronic sequence variations of the NBN gene in French Canadian breast cancer cases and healthy controls.

SNP	SNP ID^1^ (dbSNP ID)	Location	Series (No.)	MAF^2^	Common homozygote No. (expected)^3^	Heterozygote No. (expected)^3^	Rare homozygote No. (expected)^3^	HWE p-value	OR (95% CI)^4^	Reported MAF in Caucasian
10	c.-110-242delAGTA	Promoter	Cases (97)	0.082	81 (81.66)	16 (14.68)	0 (0.66)	0.38	3.4	
	(rs36226237)		Controls (72)	0.028	68 (68.06)	4 (3.89)	0 (0.06)	0.81	(1.1–10.5)	

11	c.702+149T/C	Intron 6	Cases (97)	0.082	83 (81.66)	12 (14.68)	2 (0.66)	0.07	0.7	0.108–0.117^5^
	(rs3026271)		Controls (73)	0.089	60 (60.58)	13 (11.84)	0 (0.58)	0.40	(0.3–1.6)	

12	c.703-29C/T	Intron 6	Cases (97)	0.015	94 (94.02)	3 (2.95)	0 (0.02)	0.88	1.1	0.021^5^
	(NA)^6^		Controls (73)	0.014	71 (71.01)	2 (1.97)	0 (0.01)	0.91	(0.2–7.0)	

13	c.703-18G/A	Intron 6	Cases (97)	0.026	92 (92.06)	5 (4.87)	0 (0.06)	0.79	3.9	
	(rs769418)		Controls (73)	0.007	72 (72.00)	1 (0.99)	0 (0)	0.95	(0.4–34.2)	

14	c.896+36G/A	Intron 7	Cases (97)	0.026	92 (92.06)	5 (4.87)	0 (0.06)	0.79	3.9	0.021–0.05^5^
	(rs1805826)		Controls (73)	0.007	72 (72.00)	1 (0.99)	0 (0)	0.95	(0.4–34.2)	

15	c.897-42G/C	Intron 7	Cases (97)	0.015	94 (94.02)	3 (2.95)	0 (0.02)	0.76	0.4	
	(NA)^6^		Controls (73)	0.034	68 (68.09)	5 (4.83)	0 (0.09)	0.76	(0.1–1.9)	

16	c.994+233G/A	Intron 8	Cases (97)	0.294	50 (48.37)	37 (40.25)	10 (8.37)	0.43	0.7	0.28^5^
	(rs6990969)		Controls (74)	0.311	34 (35.15)	34 (31.70)	6 (7.15)	0.53	(0.4–1.4)	

17	c.1124+18C/T	Intron 9	Cases (97)	0.278	52 (50.52)	36 (38.97)	9 (7.52)	0.45	0.7	0.09–0.306^5^
	(rs2234744)		Controls (74)	0.304	35 (35.84)	33 (31.32)	6 (6.84)	0.64	(0.4–1.4)	

18	c.1124+91C/A	Intron 9	Cases (97)	0.273	53 (51.24)	35 (38.52)	9 (7.24)	0.37	0.7	0.283–0.306^5^
	(rs1805818)		Controls (74)	0.304	35 (35.84)	33 (31.32)	6 (6.84)	0.64	(0.4–1.3)	

19	c.1125-79C/A	Intron 9	Cases (97)	0.284	52 (49.80)	35 (39.41)	10 (7.80)	0.27	0.8	0.323^5^
	(rs1805786)		Controls (72)	0.292	36 (36.13)	30 (29.75)	6 (6.13)	0.94	(0.4–1.5)	

20	c.1915-7A/G	Intron 12	Cases (97)	0.284	52 (49.80)	35 (39.41)	10 (7.80)	0.27	0.8	0.20–0.312^5^
	(rs2308962)		Controls (70)	0.300	34 (34.30)	30 (29.40)	6 (6.30)	0.86	(0.4–1.5)	

21	c.2071-30A/T	Intron 13	Cases (97)	0.284	52 (49.80)	35 (39.41)	10 (7.80)	0.27	0.7	
	(rs3736639)		Controls (71)	0.310	33 (33.82)	32 (30.37)	6 (6.82)	0.65	(0.4–1.3)	

22	c.2234+86T/G	Intron 15	Cases (97)	0.010	95 (95.01)	2 (1.98)	0 (0.01)	0.92	3.8	
	(NA)		Controls (72)	0.000	72 (72.00)	0 (0)	0 (0)	1.00	(0.2–80.3)	

23	c.2234+88C/G	Intron 15	Cases (97)	0.015	94 (94.02)	3 (2.95)	0 (0.02)	0.88	0.4	0.017–0.042^5^
	(rs13312970)		Controls (71)	0.035	66 (66.09)	5 (4.82)	0 (0.09)	0.76	(0.1–1.8)	

24	c.2234+157A/G	Intron 15	Cases (97)	0.046	88 (88.21)	9 (8.58)	0 (0.21)	0.63	2.3	0.000–0.025^5^
	(rs13312971)		Controls (71)	0.021	68 (68.03)	3 (2.94)	0 (0.03)	0.86	(0.6–8.9)	

^1 ^According to the nomenclature guidelines of the Human Genome Variation Society (reference sequence NM_002485.4), nucleotide number one being the A from the ATG codon. ^2 ^Minor allele frequency ^3 ^As expected under Hardy-Weinberg equilibrium ^4 ^Odds ratios for comparison of heterozygotes versus common homozygotes ^5 ^From NCBI dbSNP data. ^6 ^No entry in NCBI dbSNP database, although reported in the literature.

### Identification of NBN alternative splice forms

Analysis of *NBN *cDNA was also performed on a subset of these breast cancer cases and highlighted the presence of two distinct alternative splice events. The first splice variant involved the insertion of 50 bp of the intron between exons 2 and 3 and is expected to result in a premature stop codon [29]. The other alternative splice form identified involves the skipping of exons 12 to 14 which is expected to produce an in-frame deletion of 113 amino acids in the region of the NBN protein involved in the interaction with its partner MRE11A (Figure 1). However, QRT-PCR of this specific form was performed on cDNA samples from a subset of 10 breast cancer cases from our cohort and showed a very low expression relative to the main isoform (data not shown), which is consistent with previous work [30].

**Figure 2:**
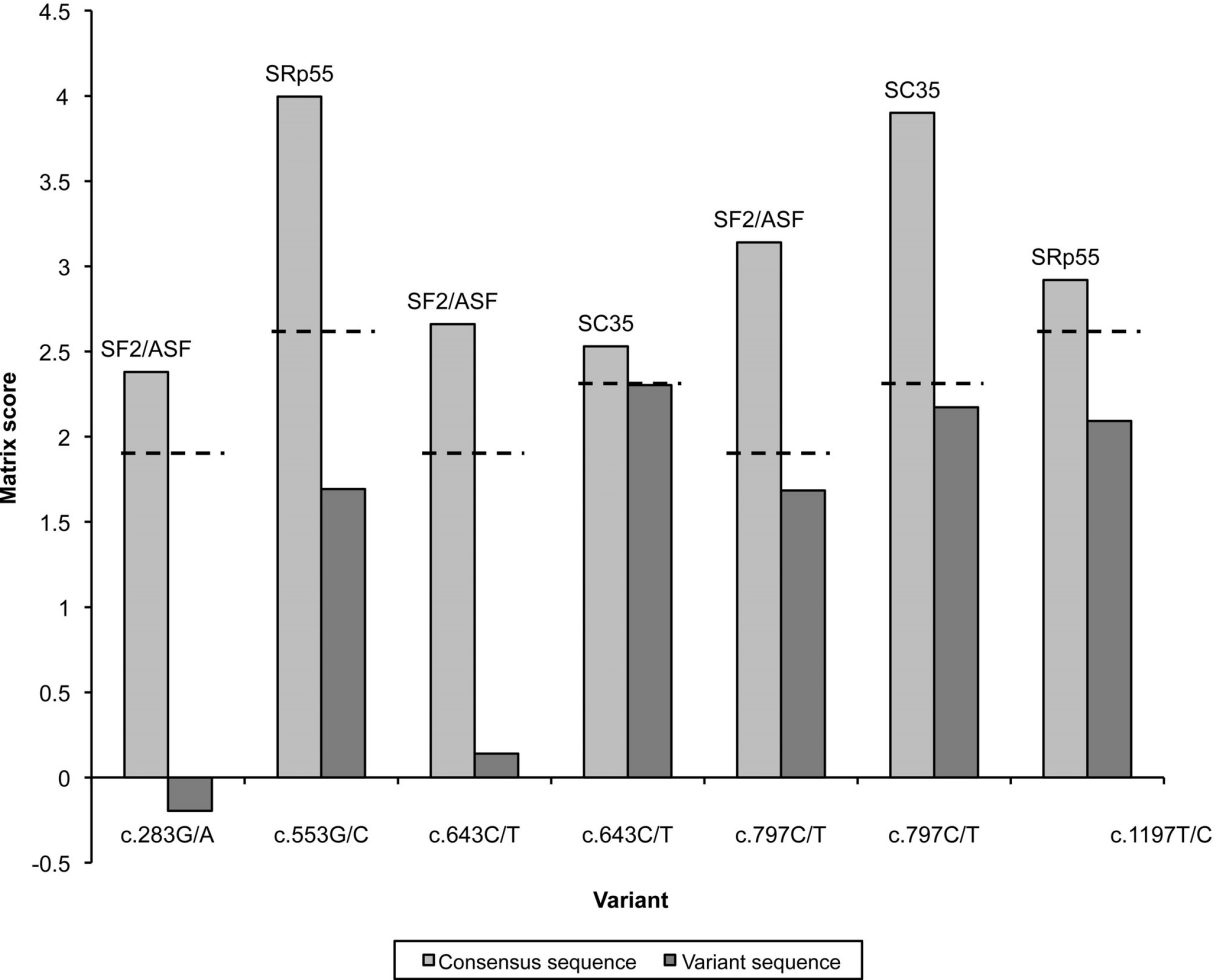
***In silico *analysis of the effect of coding variants on putative exonic splicing enhancer (ESE) motifs by the ESEfinder program**. Only scores for predicted motifs potentially abolished by exonic variants are shown. Light gray: motif matrix scores for consensus sequences. Dark gray: motif matrix scores for variant sequences. The name of the SR protein potentially involved is indicated for each score pair. Doted line indicates the default threshold used by ESEfinder for the corresponding motif.

*In silico *analysis of the putative impact of NBN exonic and intronic sequence variants on these splice events was also performed. Analysis of the nine exonic sequence variants using ESEfinder indicated that, while five variants (c.283G/A, c.553G/C, c.643C/T, c.797C/T, and c.1197T>C) might have an impact on putative score motifs of four SR proteins, a closer examination of these results shows that these scores, while below the program thresholds, remain relatively high and thus are unlikely to affect *NBN *constitutive splicing (Figure 2). According to the Splice Site prediction program, the intronic variant c.896+36G/A might slightly increase a putative acceptor site (score from 0.66 to 0.75). However, this was not confirmed by the Splice Site Finder program, which predicted the abolition of a putative donor site with a score of 71.2. As for the c.1124+91C/A intronic variant, only the Splice Site Prediction program predicted the abolition of a weak acceptor site (score of 0.45) while for the c.2234+157A/G intronic variant, the Splice Site Finder program estimated the creation of a putative acceptor site with a score of 77.2. Hence, none of the variants identified are expected to be responsible for the alternative splice events observed.

### Effect of the c.-242-110delAGTA variant on luciferase reporter gene expression

**Figure 3:**
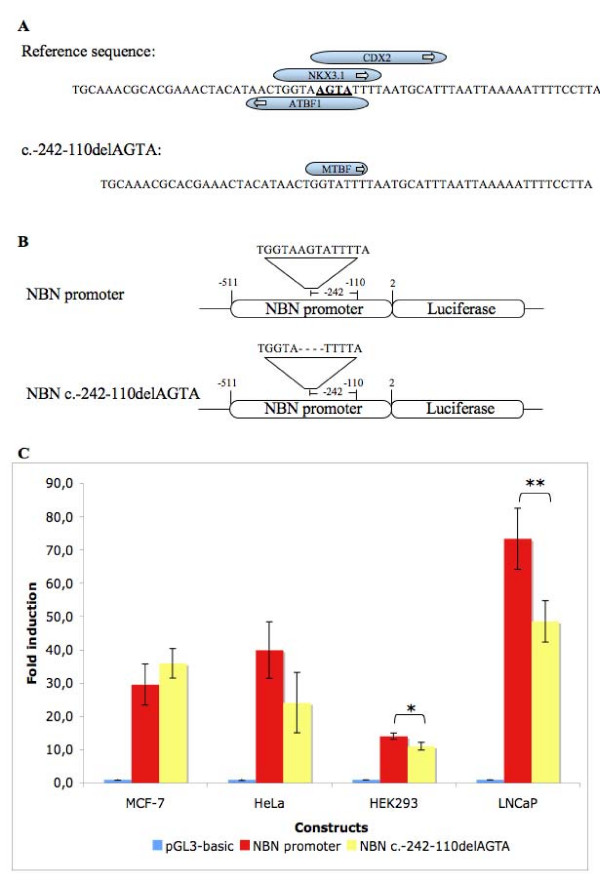
**Effect of the c.-242-110delAGTA deletion on *NBN*promoter activity**. Panel A shows the results of the *in silico *predictions of transcription factors binding sites affected by the c.-242-110delAGTA variant obtained from the MatInspector program. Panel B shows the schematic representation of the two reporter gene constructs containing the *NBN *promoter used in this study. The positions relative to the nucleotide A of the *NBN *reference sequence ATG codon are indicated. Panel C shows the results of the luciferase reporter assay. MCF-7, LNCaP, HeLa and HEK293 cells were transiently co-transfected with the *Renilla *reporter plasmid (pRL) as a transfection control. Each data represents mean ± standard deviation of triplicates. Data are shown as fold of induction compared to the activity of cells transfected with the empty pGL3-basic luciferase reporter vector. *p = 0.0245 **p = 0.0181

The putative effect of the c.-242-110delAGTA variant located in the promoter region was also assessed by the MatInspector program, which predicts that this variant may abolish recognition motifs for the ZFHX3/ATBF1 (Zinc finger homeobox 3, or AT motif-binding factor 1), NKX3-1 (NK3 homeobox 1) and CDX2 (Caudal-type homeobox 2) transcription factors, and create a potential binding site for the MTBF (Muscle-specific MT binding factor) transcription factor (Figure 3A). The effect of the c.-242-110delAGTA variant on *NBN *expression was therefore further assessed using a dual reporter gene system. MCF-7, LNCaP, HeLa and HEK293 cells were then transiently transfected with a construct containing the consensus *NBN *sequence or with the variant sequence, together with the *Renilla *reporter plasmid as an internal transfection control (Figure 3B). While no significant difference in expression was observed between both constructs in MCF-7 and HeLa cells, a slight diminution of luciferase expression was observed for the variant construct relative to the reference sequence construct in both the HEK293 and LNCaP cell lines (Figure 3C). Interestingly, QRT-PCR measures indicated that ATBF1 mRNA is 2.4 times more expressed in HeLa cells, and more than 4.3 times more expressed in LNCaP cells, than in MCF-7 cells (data not shown).

### LD analysis across NBN genomic sequence and haplotypes determination

**Figure 4:**
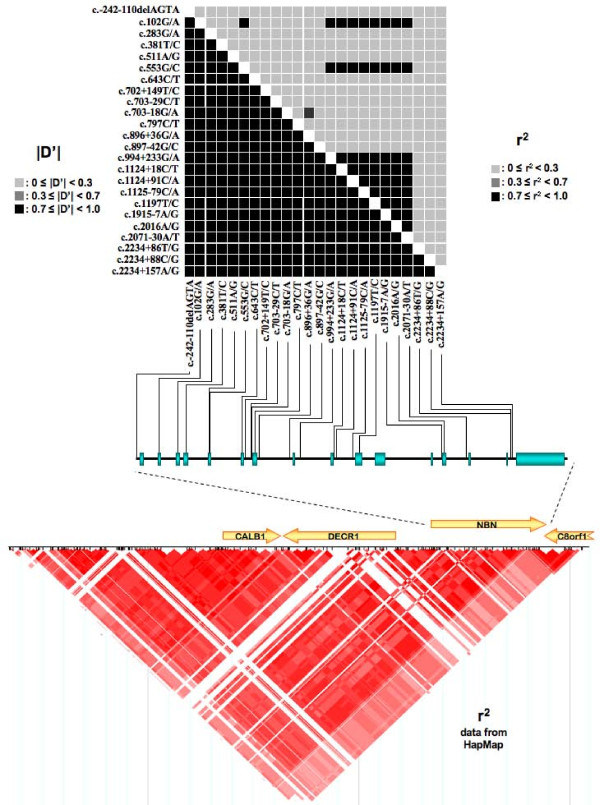
**Linkage disequilibrium across the *NBN *gene and neighbouring chromosomal region**. Upper part shows linkage disequilibrium (LD) results (r^2 ^and |D'|) obtained from the control genotypes data used in our study and their relative position along *NBN *sequence. In the bottom part of the figure are indicated r^2 ^data from the International HapMap project (release 21a) for the genomic region encompassing *NBN*. The intensity of the box color is proportional to the strength of LD. r^2 ^= 0 in white; 0 < r^2 ^< 1 in shades of pink/red; r^2 ^= 1 in bright red.

Pairwise LD measures of all variants using the control dataset are presented in Figure 4. |D'| values show a high degree of LD for all SNP pairs while the more stringent r^2 ^measure, dependent upon allele frequency, shows a limited block of LD involving mainly SNPs located in the second half of the *NBN *gene. The bottom part of Figure 4 shows the r^2 ^HapMap CEU data in the vicinity of *NBN *on chromosome 8, and suggests that *NBN *may overlap two blocks of LD. The major part of *NBN *seems to be in a strong block of LD extending 5' of the gene, while its 3' extremity spans over another smaller block.

Haplotype phasing of *NBN *using the WHAP program with all 24 variants identified in our datasets indicated that 14 haplotypes are present with an estimated frequency greater than 0.5%. Although WHAP did not estimate a significant difference between both groups, the p-value observed was borderline significant (p = 0.0588). The majority of these estimated haplotypes (84.5%) have a frequency greater than 2%. Two haplotypes (#4 and #7 in Table 4) showed a weak significant association with breast cancer, being more frequent among cases (p = 0.0205 and p = 0.0403, respectively). The same analysis, using only common variations (MAF >5%), suggested that one haplotype is more frequent among cases than controls (WH4, p = 0.0227), with another haplotype showing a non-significant trend of over-representation in cases (WH5, p = 0.0883) (Table 5). Global estimation of haplotypes was further confirmed using the PHASE 2.1.1 program, with concordant results (data not shown).

**Table 4:** Estimated haplotypes using the WHAP program and all variants identified, for cases and controls combined.

		Estimated Haplotype Frequencies			
			
Haplotype	SNPs^1^ 10-1-2-3-4-5-6-11-12-13-7-14-15-16-17-18-19-8-20-9-21-22-23-24	Cases	Controls	Combined	p-value^2^
1	TGGTAGCTCGCGGGCCCTAAATCA	0.481	0.486	0.483	0.92
2	TAGTACCTCGCGGATAACGGTTCA	0.232	0.255	0.244	0.636
3	TGGTAGCCCGCGGGCCCTAAATCA	0.065	0.097	0.078	0.29
4	AGGTAGCTCGCGGGCCCTAAATCA	0.089	0.030	0.052	0.0205
5	TAGTAGCTCGCGGATAACGGTTCA	0.036	0.007	0.022	0.147
6	TGGTAGCTCGCGCGCCCTAAATCA	0.012	0.036	0.021	0.146
7	TAGTACCTCGCGGATAACGGTTGA	0.006	0.037	0.019	0.0403
8	TGGTAGCTCGCGGGCCCTAAATCG	0.019	0.022	0.015	0.846
9	TGGTAGCTCACAGGCCCCAAATCA	0.013	0.000	0.011	0.11
10	TGGTACCTCGCGGGCCCTAAATCA	0.012	0.008	0.010	0.749
11	TGGTAGCTTGCGGGCCCTAAATCA	0.006	0.014	0.008	0.47
12	TGGTAGCTCGCGGACCCTAAATCA	0.006	0.007	0.006	0.897
13	TGGTAGCTTGCGGGCCCTAAATCG	0.011	0.000	0.006	0.126
14	TGGTAGCTCACAGGCCCCAAATCG	0.011	0.008	0.006	0.128

^1^SNPs identification as in Table 2 and 3. ^2 ^p-value for haplotype-specific test, each haplotype versus all other haplotypes.

**Table 5:** Estimated haplotypes using the WHAP program and SNPs with a frequency greater than 5% for cases and controls combined.

		Estimated Haplotype Frequencies				
				
Haplotype	SNPs^1^ 10-1-5-11-16-17-18-19-8-20-9-21	Cases	Controls	Combined	p-value^2^	OR (95%CI)
WH1	TGGTGCCCTAAA	0.511	0.558	0.538	0.361	REF
WH2	TACTATAACGGT	0.250	0.293	0.264	0.399	0.9 (0.6–1.6)
WH3	TGGCGCCCTAAA	0.065	0.096	0.080	0.277	0.7 (0.3–1.7)
WH4	AGGTGCCCTAAA	0.087	0.030	0.059	0.0227	3.2 (1.1–9.2)
WH5	TGGTGCCCCAAA	0.038	0.008	0.027	0.0883	5.1 (0.9–30.1)
WH6	TAGTATAACGGT	0.033	0.007	0.021	0.176	7.8 (0.8–30.5)
WH7	TGCTGCCCTAAA	0.017	0.008	0.011	0.542	2.4 (0.3–16.1)

^1^SNPs identification as in Table 2 and 3.^2^p-value for haplotype-specific test, each haplotype versus all other haplotypes.

A closer examination of these two haplotypes (WH4 and WH5) showed that the only variation present on WH4 is the c.-242-110delAGTA variant, which on its own shows a significant difference in frequency between both groups. Thus, this variant is likely to be responsible of the association observed as any further breakdown of this haplotype using a sliding window analysis shows that all combinations involving this variant display a significant association (data not shown). As for the WH5 haplotype carrying the c.1197T/C variant in exon 10, it does not show any significant difference in frequency between the both groups and, despite its strong linkage disequilibrium with the adjacent variants, it shows only a tendency towards significativity when present in combination with the major alleles at all other positions (Table 5).

## Discussion

Although the relevance of mutations in several DNA repair genes (*BRCA1, BRCA2, TP53, PTEN, PALB2, BRIP1*) in breast cancer susceptibility have been well established, the association of variants in other genes, potentially accounting for the remaining familial clustering, is not well defined. For example, heterozygous carriers of deleterious mutations in the *NBN *gene, in particular the Slavic founder mutation 657del5, has been associated with a 2- to 3-fold increased risk of cancer [6]. However the impact of other *NBN *variants on cancer risk is unclear. Nonetheless, studies have demonstrated an increased spontaneous chromosomal instability in cells from heterozygous carriers of *NBN *mutations [7,16]. In addition, the presence of a specific pattern of gene expression involving pathways of DNA repair and damage bypass, mitotic checkpoint and apoptosis was demonstrated, suggesting that cells from these carriers may display a much less efficient DNA repair system [31]. In this regard, a recent study by Someya et al. [32] showed a correlation between persistent radiation-induced NBN foci and both chromosomal instability and sporadic breast cancer risk. To address the possible implication of *NBN *sequence variants on breast cancer risk, we took advantage of our resource of high-risk breast cancer families drawn from the French Canadian population. In order to increase the statistical power of our investigation, one affected individual from each family was selected for analysis [33], each thoroughly screened for *BRCA1 *and *BRCA2 *mutations or large genomic rearrangements in these genes [21].

The majority of the coding variants identified in our cohort of breast cancer cases are situated in the forkhead-associated (FHA) domain and the two BRCT domains, and these domains have been demonstrated to be essential to NBN binding to the histone γ-H2AX [34,35]. One of the rare variants identified in this study, p.Ile171Val, was first described in acute lymphoblastic leukemia (ALL) patients [36]. This variant has been previously reported to be over-represented in some cancer cohorts [9,10,37,38], and has also been previously described at the homozygous state in a Japanese NBS patient affected with aplastic anemia and genomic instability [39]. Interestingly, analysis of the patient's and her father's lymphoblastoid cell lines demonstrated a higher frequency of spontaneous chromosomal aberrations compared with healthy controls (6- and 4-fold increase, respectively). These results suggest that the p.Ile171Val variation may be deleterious, also supported by the fact that this variant alters an amino acid which is conserved in species such as the chicken and the fruitfly [39]. However, the impact of the p.Ile171Val substitution remains to be clarified as a large study, including cases from Germany and the Republic of Belarus, did not find any association with breast cancer in those populations [40].

Another rare variant, p.Arg215Trp, has been considered pathogenic in previous studies based on its highly conserved position and the change introduced. Moreover, this variant has been identified in monozygotic twins compound heterozygotes for 657del5/p.Arg215Trp and affected with a severe form of NBS [41]. Nibrin^Trp215 ^appears to affect the correct nibrin function, and cells carrying this variant shows delayed DNA DSB rejoining [41]. Modelisation of the tandem BRCT domains suggests that the Arg215 residue is required for correct orientation of the BRCT domains and recognition of γ-H2AX [34]. In their experiment, nibrin^Trp215 ^seems to partially interfere with nibrin^Arg215 ^activity and may act in a co-dominant fashion. It is also interesting to note that although this variant is relatively frequent in several studies, to our knowledge no NBS cases homozygous for this variant have been reported.

Of the other rare variants present in our cohort of breast cancer cases, the p.Pro266Leu variant was observed in one breast cancer case, and the p.Asp95Asn variant was found in one breast cancer case and one control. Although these variants involve conserved residues, their putative effect on protein function remains unclear. In a previous study on non-Hodgkin lymphoma, both variants were detected only in healthy controls [42]. Regarding the p.Asp95Asn variant first identified in an ALL patient [36], no association was found in a study of larynx cancer [38] or ALL [37]. This variant was found in one out of 613 and 121 unselected and familial prostate cancer cases, respectively, but not in controls [43]. However, p.Asp95Asn is not predicted to be highly damaging as its expression in NBS cells seems to have a similar activity to the wild type protein [42]. The only other non-synonymous change identified in our cohort is the p.Glu185Gln common variant (MAF >25%), which has been genotyped in several association studies. Although the majority of the studies analyzing this variant found no association with cancer [12,38,42-44], some studies reported an association with breast cancer [8], basal cell carcinoma in men [45] and a recent meta-analysis of this variant in bladder cancer revealed a significant association after controlling for potential bias [46]. In addition, Musak et al. [47] reported that this variant may modulate the frequency of chromatid-type aberrations among tire plant workers. However, our data do not allow us to confirm any positive association.

Among the non-coding variants, a possible association of the c.-242-110delAGTA variant with an increased risk of breast cancer could be observed (OR 3.4 95% CI: 1.1–10.5). This association was further confirmed by the estimation of the haplotype diversity in our dataset, which highlighted the presence of the haplotype bearing the c.-242-110delAGTA variant at a higher frequency among the breast cancer case subset. *In silico *prediction of putative transcription factor binding sites indicated that of the four transcription factor binding sites potentially affected by the deletion, some are predicted to involve proteins not expressed in the breast. MTBF acts in the muscle regulation of the myostatin gene [48], while CDX2 is mainly expressed in the gut although its ectopic expression was also reported in cases of non-gastrointestinal carcinomas [49]. As for the transcription factor NKX3-1, it is mainly expressed in the prostate although it is also found at low levels in the mammary gland and breast tumors, and has been suggested to act as a haplo-insufficient tumor suppressor in prostate cancer [50]. The fourth transcription factor potentially affected is ATBF1 (also known as ZFHX3). ATBF1 was shown to suppress the expression of the alpha-fetoprotein [51] and the MYB oncogene [52]. ATBF1 also cooperates with p53 to activate the *CDKN1A *promoter and trigger cell cycle arrest [53]. ATBF1 is expressed in the mammary gland and breast tumors (NCBI's UNIGENE data), and the expression of ATBF1 mRNA was correlated with a better prognosis in 153 patients with invasive carcinomas of the breast [54]. This might suggest that deregulation of genes controlled through ATBF1 could have an important impact on breast cancer formation and progression.

Luciferase reporter gene assay using the promoter sequence proximal to the ATG codon indicated a similar level of expression of the c.-242-110delAGTA variant allele as compared to the reference sequence construct in the breast cancer cell line MCF-7 and in HeLa cells. However, a slight decrease in luciferase expression was observed in both HEK293 and LNCaP cells, which might indicate that this variant could have an impact on *NBN *expression in a cell type manner. The analysis of *ATBF1 *gene expression indicated that this transcription factor is weakly expressed, in particular in the MCF-7 cells. This is comparable to a recent study performed on a panel of 32 breast cancer cell lines, which indicated that in 75% of these cell lines, *ATBF1 *is expressed at 50% or less relative to the normal breast [55]. This could in turn explain the lack of effect observed for the luciferase assay in the MCF-7 cell line. On the other hand, one could hypothesize that *NBN *(or transcription factors affecting its expression) may be regulated following irradiation or other genotoxic stress. Additional research will however be needed to address this possibility, as well as binding experiments to confirm ATFB1 binding to this region of the promoter. However, it is also important to keep in mind that the promoter sequence cloned here, although supporting *NBN *expression, is unlikely to constitute the entire *NBN *promoter. It is indeed plausible that transcription factors acting on the proximal sequence might be influenced by other transcription factors acting further upstream. As such, despite the lack of variation in expression induction found in MCF-7 by luciferase assay, it is possible that the c.-242-110delAGTA variant could be in LD with another functional variant located elsewhere in the promoter, as a high degree of LD is present across the whole *NBN *gene. Unfortunately, the promoter variant could not be associated by LD with another variant in the coding region of the gene, which would have allowed to directly measure *NBN *allelic expression.

## Conclusion

Our analysis suggests that the variant c.-242-110delAGTA identified in our cohort of breast cancer individuals and located in the promoter region of the *NBN *gene may be associated with an increased risk of breast cancer. However, the functional analysis by luciferase promoter assay does not support a causative effect of this variant in the breast cancer cell line MCF-7, although a reduction of luciferase expression driven by the *NBN *promoter can be observed in two other cell lines tested. As the *NBN *genomic region displays a high degree of LD, we cannot rule out the effect of other variants located upstream of the region analyzed here. Alternatively, the effect of this variant may be dependent upon induction by a genotoxic stress such as irradiation. Although NBS is a rare syndrome, carriers of deleterious alterations in this gene are more common and may display subtle manifestations in cellular pathways predisposing *NBN*-mutated carriers to malignancy. Further analyses will therefore be needed to ascertain the impact of rare variants and promoter variants of *NBN *on breast cancer susceptibility in other populations.

## Abbreviations

AIR: ATM interacting region; ALL: Acute lymphoblastic leukemia; ATBF1: AT motif-binding factor 1; BRCT: BRCA1 C-terminal; CDX2: Caudal-type homeobox 2; CI: Confidence interval; DSB: Double strand breaks; ESE: Exonic splicing enhancer; FHA: Forkhead-associated; HWE: Hardy-Weinberg equilibrium; LD: Linkage disequilibrium; MAF: Minor allele frequency; MIR: MRE11A interacting region; MTBF1: Muscle-specific MT binding factor; NBS: Nijmegen breakage syndrome; NKX3-1: NK3 homeobox 1; OR: Odds ratio; QRT-PCR: Quantitative real-time PCR; ZFHX3: Zinc finger homeobox 3.

## Competing interests

The authors declare that they have no competing interests.

## Authors' contributions

SD participated in the molecular analysis study, performed the reporter gene assays experiments and bioinformatics analyses, and drafted the manuscript. CJB participated in the molecular analysis study. YL provided technical assistance and helped draft the manuscript. GO performed DNA and RNA extractions, cell maintenance and participated in the luciferase assays experiments. FD conceived the study, its design and coordination, and authored the final version of the manuscript. All authors read and approved the final manuscript.

## Pre-publication history

The pre-publication history for this paper can be accessed here:

http://www.biomedcentral.com/1471-2407/9/181/prepub
